# Assessment of Anti-Tumor potential and safety of application of Glutathione stabilized Gold Nanoparticles conjugated with Chemotherapeutics

**DOI:** 10.7150/ijms.40827

**Published:** 2020-03-12

**Authors:** Karol P. Steckiewicz, Ewelina Barcinska, Katarzyna Sobczak, Ewelina Tomczyk, Michał Wojcik, Iwona Inkielewicz-Stepniak

**Affiliations:** 1 Chair and Department of Medical Chemistry, Medical University of Gdansk, Debinki street 1, 80-211 Gdansk, Poland.; 2 Faculty of Chemistry, University of Warsaw, Pasteura 1, 02-093 Warsaw, Poland.

**Keywords:** Gold nanoparticles, Cancer, Drug deliver platform, Gold nanoparticles conjugate, Chemotherapeutic

## Abstract

Due to the high toxicity of currently used chemotherapeutics, novel methods of cancer treatment are needed. Gold nanoparticles (AuNPs) seem to be an interesting alternative due to penetration through biological membranes and systemic barriers. AuNPs as carriers of chemotherapeutics allow for reduced concentrations whilst maintaining the expected effect, and thus reducing the costs of therapy and adverse effects. We synthesized AuNPs stabilized with reduced glutathione (GSH) and conjugated with doxorubicin (DOX), gemcitabine (GEM) or cytarabine (CTA). This is the first study in which cytarabine-AuNPs were synthesized and characterized. Transmission electron microscopy (TEM), thermogravimetric analysis (TGA), nuclear magnetic resonance spectroscopy (NMR) and high-performance liquid chromatography (HPLC) were used to chemically characterize obtained nanoparticles. Antitumor activity and safety of application were assessed by MTT assay in *in vitro* model (human osteosarcoma cells -143B, human osteoblast- hFOB1.19, breast cancer cells - MCF7, breast epithelial cells - MCF10A, pancreatic cancer cells - PANC-1, and pancreatic cells - hTERT-HPNE cells). We have shown that cellular response varies according to the type and concentration of AuNPs. At some concentrations, we were able to show selective cytotoxicity of our AuNPs conjugates only to cancer cell lines. Synthesized nanoparticles were more cytotoxic to tumor cell lines than chemotherapeutics alone.

## Introduction

Cancer is one of the leading causes of death and emerging epidemiological problem in clinical practice furthermore cancer treatment is expensive 1,2. AuNPs, that have established a role in nanotechnology, may be a possible answer to that matter. It has been proven that AuNPs may be used in cancer diagnostic and therapy 3,4. They can penetrate through cellular membranes, which is crucial for biomedical applications 5. Furthermore, AuNPs as drug delivery platforms can accumulate in the cancer microenvironment, which protects healthy tissue 6. Importantly, AuNPs can be efficient in drug-resistant neoplasm 7. Another advantage of the usage of AuNPs is a variety of sizes and possible surface modifications, which greatly expands their clinical application 8-10. AuNPs have also good safety-profile 11. Furthermore, it has been shown that GSH stabilized AuNPs have good biocompatibility profile and low immunogenicity 12. Moreover, GSH as a peptide present in each cell can increase the biocompatibility of possessed AuNPs, which will be beneficial for potential applications 13.

Pancreatic cancer, osteosarcoma, and breast cancer are types of cancer were different oncological approaches may be used. Pancreatic cancer is associated with poor prognosis (6% of 5-years survival rate) 14. There are several treatment options for this cancer: surgery, radiotherapy, chemotherapy or combination of those 14. Among the others, gemcitabine (GEM) is used in chemotherapy for pancreatic cancer 14. Unfortunately, GEM has severe side-effects, among the others: nausea, mielosuppression, liver damage or heart failure which may decrease patient quality of life 15. Pancreatic cancer is thought to become the second most popular type of cancer in 2030 and the average cost of lifetime treatment per patient is as high as $65335 16,17. Another cancer considered with poor outcome is osteosarcoma, which is one of the most common cancers of the youth 18. In general treatment options are similar to pancreatic cancer (surgery, chemotherapy, radiotherapy) 18. Treatment of osteosarcoma in many cases requires amputation, which severely decreased the patient's quality of life. One of the agents used in osteosarcoma therapy is doxorubicin (DOX) 18. Also, it has been proven that AuNPs may be cytotoxic to osteosarcoma cell lines 19. For breast cancer apart from the “classical” treatment, we can use hormone-blocking therapy and monoclonal antibodies against described molecular targets. DOX and cytarabine (CTA) are effective drugs in breast cancer therapy 20,21. Unfortunately, both drugs have serious aftereffects. For DOX it will be mielosuppression, cardiotoxicity hair loos and others 22. Usage of CTA can lead to brain damage, mielosuppression, gastrointestinal tract disturbances and others 23. Surgery with healthy tissue margin typically gives the best treatment outcome; however, it is limited only to small tumors. Thus, chemotherapy still is one of the main treatment strategies in oncology. However, due to multidrug resistance of cancer and poor penetration of active agents through the tumor, its effectiveness is limited. Thus, novel drug delivery platforms, such as AuNPs, may be an interesting solution to this problem.

In this research we assessed the anti-tumor potential of GSH stabilized AuNPs conjugated with chemotherapeutics (DOX, CTA, GEM). As chemotherapeutics have severe side effects and limited effectiveness we tried to overcome their disadvantages by using AuNPs as drug delivery platforms. Our modification allows using a smaller concentration (dose) of a drug, which will increase patients' comfort, also better penetration may upsurge the efficiency of treatment.

Effectiveness against pancreatic adenocarcinoma, breast cancer, and osteosarcoma cell lines was evaluated. We also assessed the safety of AuNPs application by *in vitro* cytotoxicity assay on non-transformed cell lines.

## Materials and methods

### Synthesis

#### Synthesis of AuNPs stabilized with GSH

93.5 μL (0.135 mmol) of 30% chloroauric acid solution (HAuCl_4_) was diluted using 26 mL of distilled water. The reaction mixture was placed in a water/ice bath and then 162 mg (0.537 mmol) of reduced glutathione was added slowly in small portions. The solution turned from light yellow through brown into transparent with white suspension. After 1.5 hours a few drops of a saturated solution of sodium bicarbonate was added, which caused an increase of pH and consequently disappearance of the precipitate. Next, 50 mg (1.322 mmol) of NaBH_4_ dissolved in 6.5 mL of water was added quickly with high-speed magnetic stirring. The solution turned brown and the reaction mixture was stirred an additional 2 hours. Then, 20 mL of methanol was added to precipitate nanoparticles. The precipitate was centrifuged (5000 rpm, 10 min) washed with methanol: water 1:1 mixture and dissolved in 1 mL of distilled water. The significant concentration of nanoparticles left in supernatant and was subjected to additional precipitation. The obtained supernatant was mixed with 55 mL of methanol and was centrifuged (10000 rpm, 15 min.) yielding brown sediments. The precipitate was washed with methanol: water 4:1 mixture and was dried. The additional precipitate was used in this study. Then nanoparticles were precipitated an additional two times. Resulted precipitates were left to dry in the air.

#### Synthesis of AuNPs stabilized with GSH and DOX

4 mg of AuNPs stabilized with GSH were dissolved in 0.4 mL of distilled water. Then, 2 mL of prepared DOX water solution (1 mg/mL) was added slowly. The reaction mixture was stirred 12 hours and then nanoparticles were purified using centrifugal filters (Amicon® Ultra 0.5 mL) in a centrifuge (10000 rpm, 5 min) and washed two times with PBS.

#### AuNPs stabilized with GSH and GEM

20 mg of AuNPs stabilized with GSH were dissolved in 2 mL of distilled water. Then, 1 mg (0.003 mmol) of GEM dissolved in 1 ml of water was added and the reaction was mixed for 12 hours. Next, nanoparticles were purified using centrifugal filters (Amicon® Ultra 0.5 mL) in a centrifuge (10000 rpm, 5 min).

#### AuNPs stabilized with GSH and CTA

20 mg of AuNPs stabilized with GSH were dissolved in 2 mL of distilled water. Then 1 mg (0.004 mmol) of CTA dissolved in 1 mL of water was introduced and the reaction was mixed for 12 hours. Next, nanoparticles were purified using centrifugal filters (Amicon® Ultra 0.5 mL) in a centrifuge (10000 rpm, 5 min).

### Cell culture

143B (ATCC CRL-8303) were cultured in Minimum essential medium (Eagle) with 0.015 mg/mL 5-bromo-2'-deoxyuridine. Media was supplemented with 10% of heat-inactivated fetal bovine serum (FBS) and 1% of penicillin and streptomycin (P/S) hFOB 1.19 (ATCC CRL-11372) were cultured in 1:1 mixture of Ham's F12 Medium and Dulbecco's Modified Eagle's Medium (DMEM/F12) with 2,5 mM of L-glutamine. The media was supplemented with 10% of FBS and 1% of P/S. hTERT-HPNE (ATCC CRL-4023) were cultured in DMEM with 2 mM of L-glutamine and Medium M3 base in ratio 3:1. Media was supplemented with 5% of FBS, 10 ng/mL of EGF, 1g/L of D-glucose, 750 ng/mL of puromycin and 1% of P/S. MCF 10A (ATCC HTB-22) were cultured in DMEM/F12 media supplemented with 5% of horse serum, 20 ng/mL of EGF, 0,5 mg/mL of hydrocortisone, 100 ng/mL of cholera toxin, 10 g/mL of insulin and 1% of P/S. MCF 7 (ATCC CRL-10317) were cultured in DMEM (4mM L-glutamine and 4500 mg/L of glucose). The media was supplemented with 10% of FBS and 1% of P/S. PANC 1 (ATCC CRL-1496) were cultured in DMEM (4mM L-glutamine and 4500 mg/L of glucose). The media was supplemented with 10% of FBS and 1% of P/S. All cells were possessed from the American Type Culture Collection. Cells were kept in T-75 flask under the sterile condition at 37°C in a humidified atmosphere of 5% of CO_2_ (medium renewal every 2 days). When confluent cells were detached with a trypsin-EDTA solution and subcultivated according to ATCC guidelines.

### Treatments

Each time just before experiment new dilutions of synthesized AuNPs in FBS-free media were prepared. The stock solution was shaken well to ensure an equal dispersion of AuNPs. Cells were incubated with 1, 10, 25, 50 and 100 µg/mL of all synthesized nanoparticles (AuNPs-GSH, AuNPs-GSH-GEM, AuNPs-GSH-DOX, AuNPs-GSH-CTA). Prior to incubation solutions were shaken in order to prevent agglomeration of investigated AuNPs. Control cells were kept in FBS-free media without AuNPs addition. Cells were incubated in 37°C, 5% CO_2_ for 24h.

### MTT assay

Cell viability was measured by MTT assay with a previously established method 19,24. Briefly, cells were seeded into a 96-well dish (density 1x10^4^ cells/well). After 24h cells were incubated with synthesized AuNPs and chemotherapeutics as described in the ''Treatments” section. After 24h solution water-soluble tetrazolium salt was added to a final concentration of 0.5 mg/mL. Next, the plate was incubated for 2h in standard condition. Formazan crystals were diluted in dimethyl sulfoxide. Cell viability was assessed by absorbance measurements. Absorbance values were adjusted with blank NPs. The viability of control cells was set to 100%.

### Statistical analysis

All statistical analysis was performed with GraphPad Prism 5 software (GraphPad Software, Inc., USA). Statistical analysis was determined by a one-way analysis of variance (ANOVA) and Tukey's posthoc test.

## Results

### AuNPs characteristic

#### AuNPs stabilized with GSH (AuNPs-GSH)

**Figure 1:**
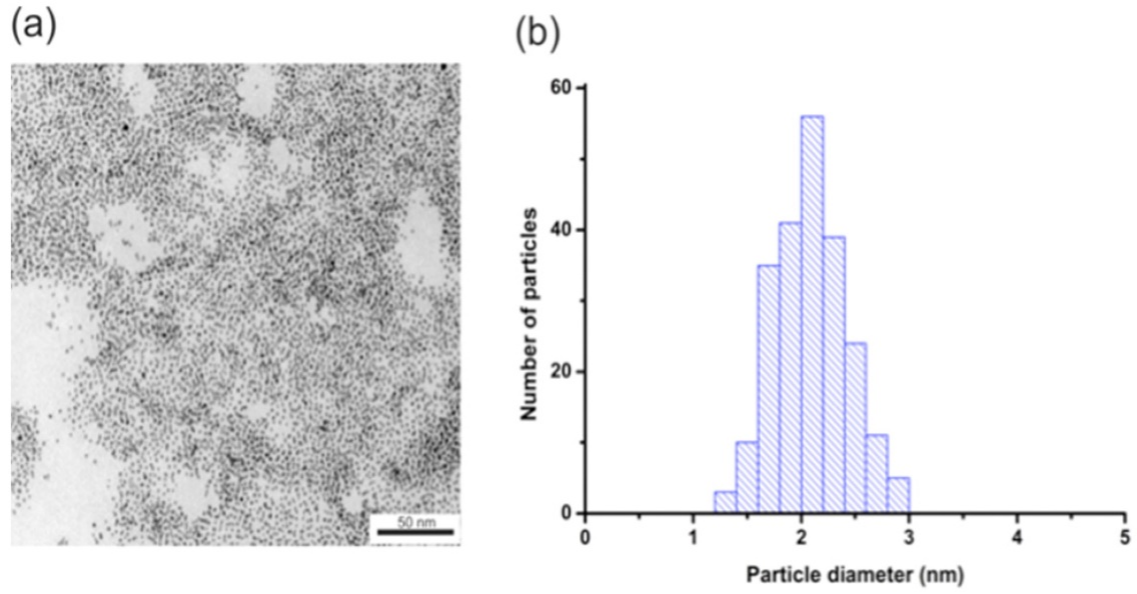
(a) AuNPs-GSH TEM image and (b) AuNPs-GSH histogram of size distribution. Scale bar corresponds to 50 nm.

To determine the size and monodispersity of obtained nanoparticles we conducted TEM measurements. Figure 1 shows the TEM image of AuNPs (Figure 1a) and histograms of the size distribution (Figure 1b). The obtained nanoparticles have an average diameter of about 2.1 +/- 0.3 nm.

**Figure 2:**
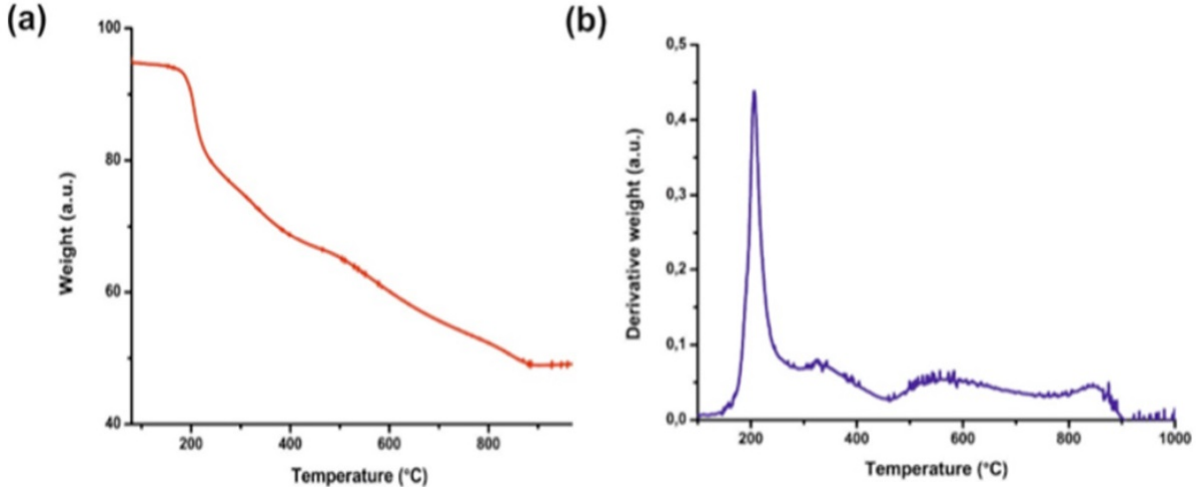
TGA analysis of AuNPs-GSH: (a) TGA curves of the studied sample and (b) the first derivative of its weight loss.

The presence of GSH on the surface of nanoparticles has been confirmed by thermogravimetric analysis. The given thermogram (Figure 2) shows the weight loss of the sample during heating (red line). The first derivative of TGA curve (blue line) shows a single sharp peak in the temperature range 220-250°C. It corresponds to the rapid decomposition of nanoparticles as a result of the loss of GSH from the surface of nanoparticles. This analysis showed that GSH is ~24% of the mass of AuNPs.

**Figure 3:**
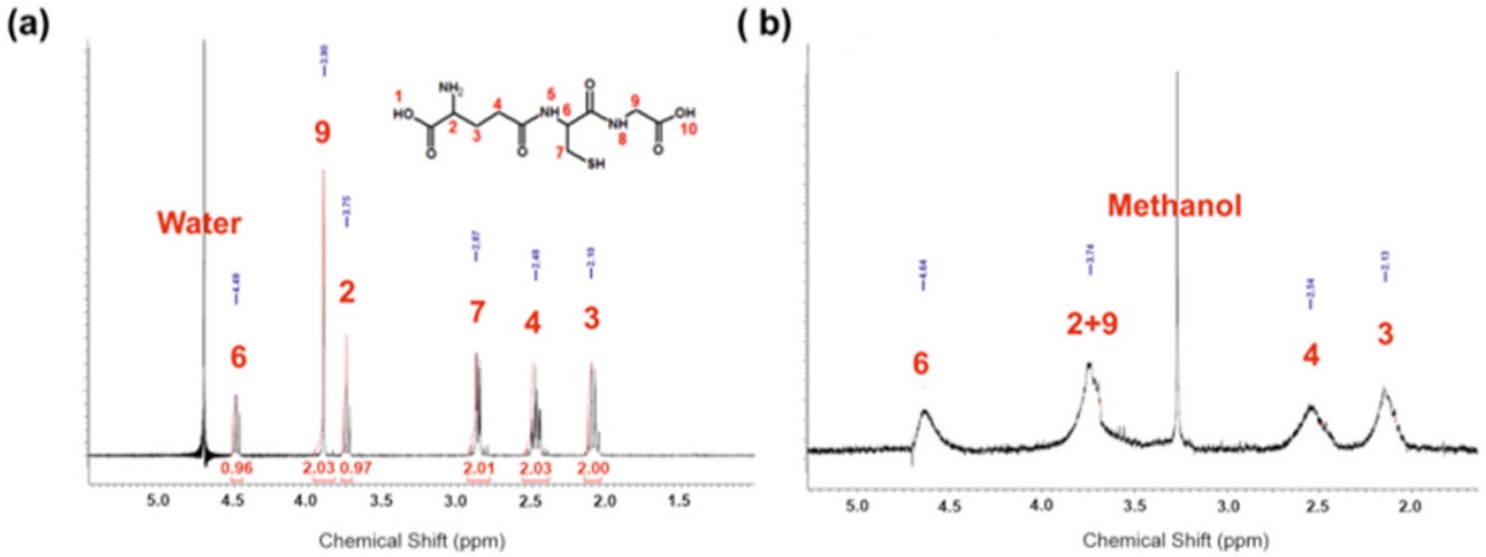
(a) 1H NMR of AuNPs-GSH (b) simulation of 1H NMR spectra of GSH.

The presence of GSH on the surface of the nanoparticles was also confirmed by ^1^H NMR spectra (Figure 3a), for comparison spectra of GSH is shown in Figure 3b. In Figure 3a there are clearly visible four signals characteristic for GSH (strong signal 3.42 ppm arises from methanol). All signals are broadened which is characteristic for NMR spectra protons from molecules conjugated to the surface of nanoparticles (no sharp signals indicate that sample was purified properly and there is no unbounded GSH). Signals at 2.30 ppm, 2.69 ppm, and 3.89 ppm are assigned respectively to protons from carbons 3, 4 and 9. The broad signal at 3.89 ppm is probably a screening signal from proton from carbon 2, which should appear at 3.84 ppm. Protons from carbons 6 and 7 are in β and α position to a thiol group, which is located in direct neighborhood to gold atoms, which cannot be analyzed by 1D spectra.

#### AuNPs stabilized with GSH and DOX (AuNPs-GSH-DOX)

**Figure 4:**
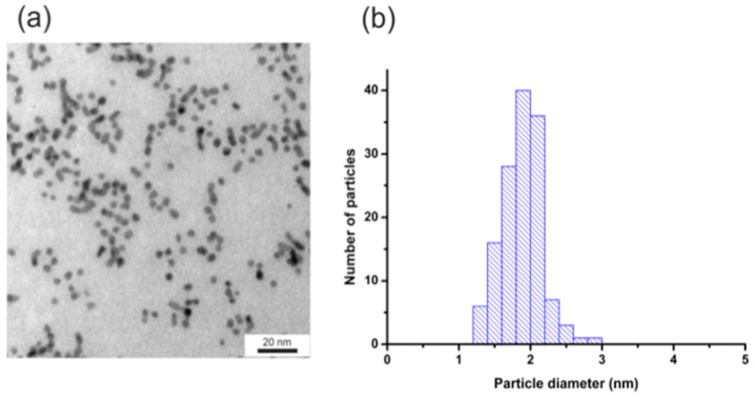
(a) TEM image and (b) histogram of NPs size distribution for AuNPs-GSH-DOX. Scale bar corresponds to 20 nm.

Figure 4 shows the TEM image (Figure 4a) and the histogram of the size distribution (Figure 4b) of nanoparticles. AuNPs-GSH-DOX have an average diameter of about 1.9+/- 0.3 nm after the surface modification. The lower diameter value of these nanoparticles is related to lower contrast characteristics for samples with higher organic fraction concentration.

**Figure 5:**
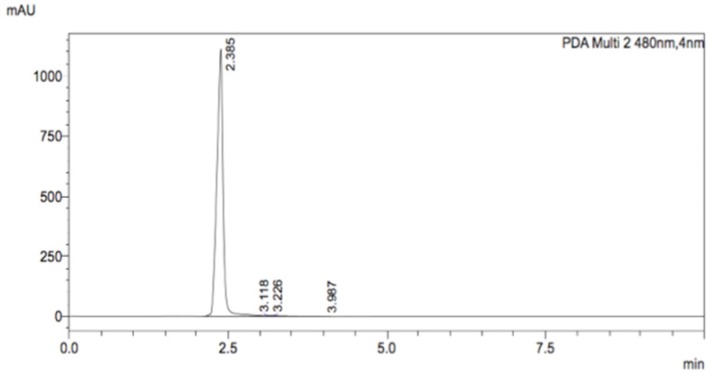
HPLC chromatogram of decomposed AuNPs-GSH-DOX solution.

Sample of these nanoparticles was treated with iodine in order to break bonds between nanoparticles and ligands. Ligands solution was separated from nanoparticles aggregates and HPLC measurement was performed. The mobile phase consisted of 10 mM KH_2_PO4 and 0.1% (v/v) trifluoroacetic acid in water and acetonitrile as the organic phase. A gradient method was used in which the mobile phase started as 75% aqueous phase and 25% of the organic phase and changed in a linear manner to 60/40 within 7 minutes. Peaks were monitored using a UV-VIS detector. Figure 5 shows a strong signal (2.385 min), which is characteristic of DOX.

#### AuNPs stabilized with GSH and GEM (AuNPs-GSH-GEM)

**Figure 6:**
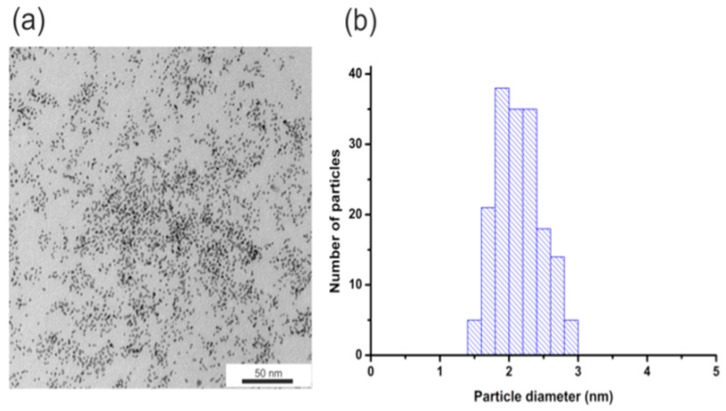
(a) TEM image and (b) histogram of NPs size distribution for AuNPs-GSH-GEM. Scale bar corresponds to 50 nm.

Figure 6 shows the TEM image (Figure 6a) and the histogram of the size distribution (Figure 6b). The obtained nanoparticles have an average diameter of about 2.11+/- 0.33 nm.

#### AuNPs stabilized with GSH and CTA (AuNPs-GSH-CTA)

**Figure 7:**
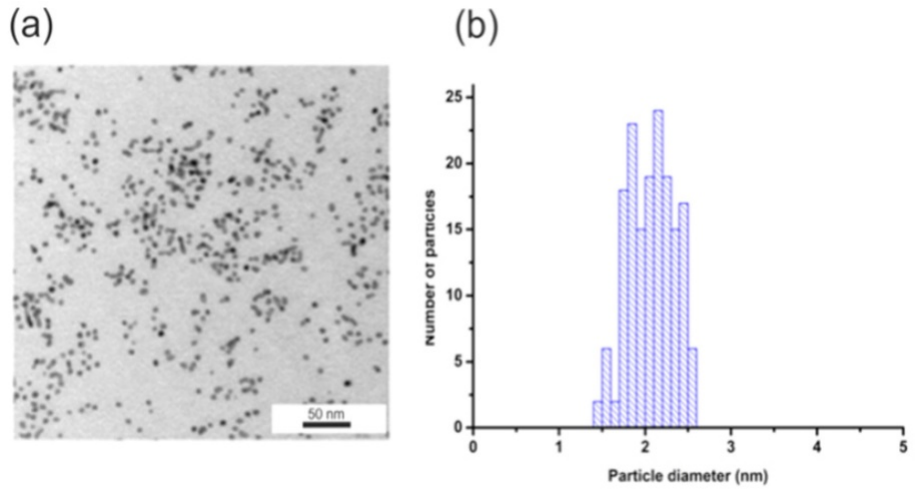
(a) TEM image and (b) histogram of NPs size distribution for AuNPs-GSH-CTA. Scale bar corresponds to 50 nm.

Figure 7 shows the TEM image (Figure 7a) and the histogram of the size distribution (Figure 7b). AuNPs-GSH-CTA have an average diameter of about 2.10+/- 0.35 nm. The lower diameter value of these nanoparticles is related to lower contrast characteristics for samples with higher organic faction concentration.

### AuNPs decreased the viability of the cells in a concentration-dependent manner

**Figure 8:**
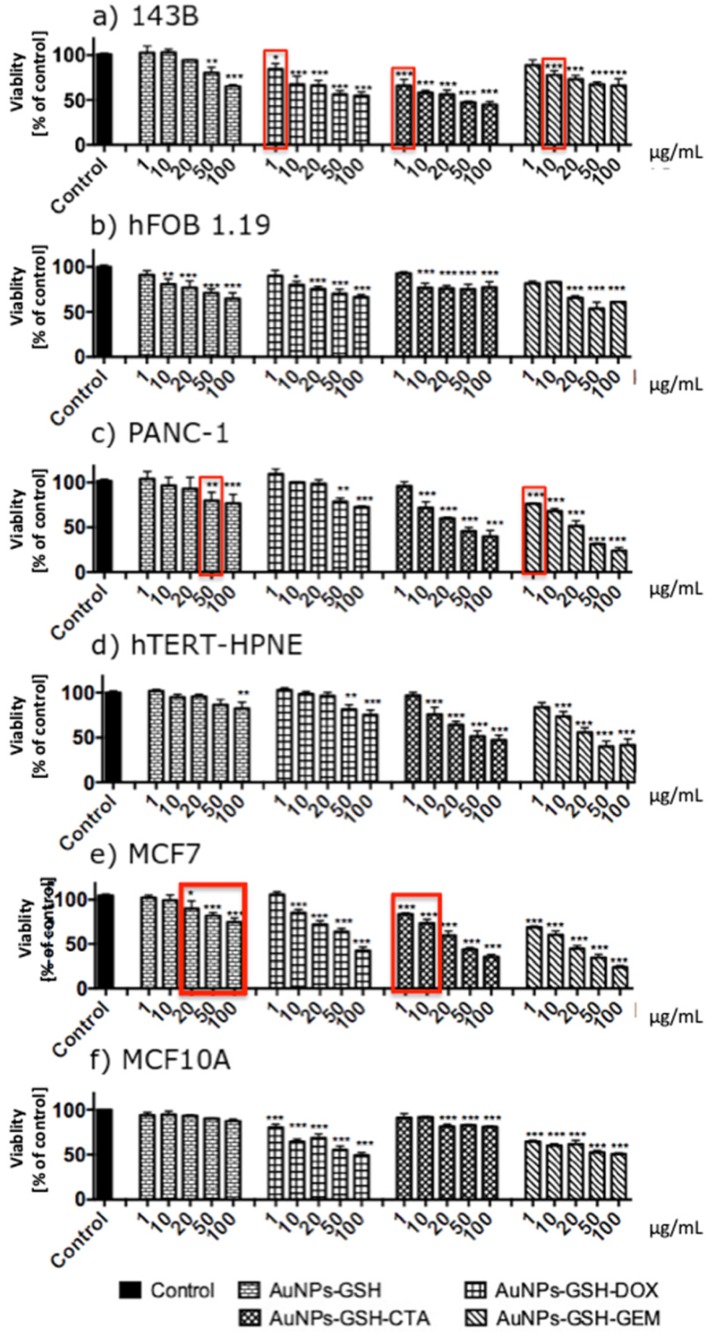
AuNPs-GSH conjugated with chemotherapeutics: DOX, GEM, CTA decreased viability of the cells in a concentration-dependent manner after 24 h of incubation. The viability measured by MTT assay of (a) 143B, (b) hFOB 1.19, (c) PNAC-1, (d) hTERT-HPNE, (e) MCF7 and (f) MCF10A cells exposed to AuNPs for 24h. Data are presented as mean ±SD. *p<0.05, **p<0.01, ***p<0.001. Red boxes indicate selective cytotoxicity of AuNPs only to cancer cells (in comparison to non-transformed cells).

AuNPs-GSH-DOX and AuNPs-GSH-GEM and all AuNPs-GSH-CTA decreased the viability of 143B cells. All tested AuNPs decreased the viability of hFOB 1.19 cells. The highest impact on 143B cells viability had AuNPs-GSH-CTA; this AuNPs in the concentration of 100 µg/mL demonstrated approximately 45% decreased viability of 143B cells. In contrast, the highest impact on the viability of hFOB 1.19 cells had AuNPs-GSH-GEM (in the concentration of 100 µg/mL). Interestingly, in lower concentrations (1, 10 µg/mL) hFOB 1.19 were more susceptible to AuNPs than 143B cells (Figure 8).

All tested AuNPs decreased the viability of PNAC-1 and hTERT-HPNE cells in a concentration-dependent manner. Importantly, hTERT-HPNE cells were more resistant to AuNPs than PANC-1 cells. The highest impact on PANC-1 viability had AuNPs-GSH-GEM. AuNPs-GSH-CTA to around 30% and AuNPs-GSH-GEM decreased the viability of the cells to around 25%. Similarly for hTERT-HPNE AuNPs-GSH-CTA and AuNPs-GSH-GEM decreased the viability of the cells to around 45% (Figure 8).

All tested AuNPs decreased the viability of MCF7 cells and all tested AuNPs apart from AuNPs-GSH decreased viability of MCF10A cells. MCF10A were more resistant to AuNPs than MCF7 cells. The highest impact on MCF7 cells viability had AuNPs-GSH-GEM (decreased viability to around 25%). AuNPs-GSH-DOX and AuNPs-GSH-GEM in 100 µg/mL concentrations decreased viability of MCF10A cells to 50% (Figure 8).

We have proven that AuNPs-GSH-DOX in concentration 1 µg/mL, AuNPs-GSH-CTA in concentration 1 µg/mL and AuNPs-GSH-GEM in concentration 10 µg/mL are selectively cytotoxic to osteosarcoma cell line (143B) in comparison to non-transformed cells. AuNPs-GSH-GEM in concentration 1 µg/mL is selectively cytotoxic against pancreatic ductal adenocarcinoma cells (PANC-1). AuNPs-GSH-CTA in concentration 1 and 10 µg/mL is selectively cytotoxic to epithelial breast adenocarcinoma cells (MCF7).

Generally, cancer cell lines (143B, PANC1, MCF7) were more susceptible to our conjugates that non-transformed cell lines (hFOB1.19, hTERT-HPNE, MCF10A). In Figure 8 red boxes indicate selective cytotoxicity of AuNPs only to cancer cells (in comparison to non-transformed cells).

**Figure 9:**
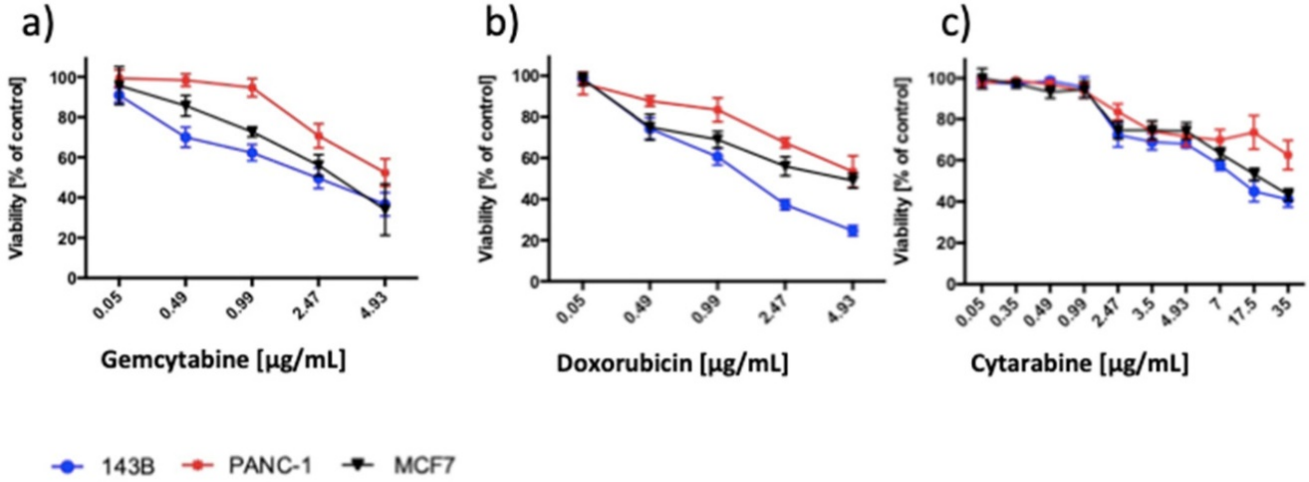
Chemotherapeutics decreased the viability of the cancer cells in a concentration-dependent manner. Viability measured by MTT assay of the 143B, PANC-1 and MCF7 cells exposed to (a) GEM, (b) DOX and (c) CTA for 24h. The viability of control was set to 100%. Data are presented as mean ± SD.

As a reference, we assessed the impact of chemotherapeutics on the viability of cancer cell lines (Figure 9). DOX in a concentration equal or higher 0.49 µg/mL decreased the viability of 143B and MCF7 cells and in a concentration equal or higher 0.99 µg/mL of PANC1 cells. CTA decreased the viability of 143B, PANC-1 and MCF7 cells in concentrations equal to or higher than 2.47 µg/mL. GEM significantly decreased the viability of 143B cells in a concentration equal or higher 0.49 µg/mL, PANC-1 2.47 µg/mL and MCF7 0.99 µg/mL.

In the conditions listed below, our AuNPs-chemotherapeutic conjugates significantly decreased the viability of cancer cells, whereas corresponding chemotherapeutic concentrations did not. AuNPs-GSH-DOX (1 µg/mL), AuNPs-GSH-CTA (1, 10, 20, 50 µg/mL) on 143B cells; AuNPs-GSH-CTA (10, 20, 50 µg/mL), AuNPs-GSH-GEM (1, 10, 20 µg/mL) on PANC-1 cells; AuNPs-GSH-CTA (1, 10, 20, 50 µg/mL) and AuNPs-GSH-GEM (1, 10 µg/mL) on MCF7cells, respectively.

Importantly, tested AuNPs had anti-cancer potential. Nanoparticles coated only with GSH had the smallest impact on the viability of the cells. Furthermore, tumor cells line were more susceptible to tested AuNPs that non-transformed cell lines. As mentioned above AuNPs conjugated with chemotherapeutics exerted selective cytotoxicity.

## Discussion

In 2018, more than 18 million people were diagnosed with cancer and 9.5 million people died of it 25. Among cancers: breast cancer (>2 088 000 new cases, >626 000 deaths in 2018), pancreatic cancer (>458 000 new cases, 432 000 deaths in 2018) and osteosarcoma (morbidity rate of 4 cases/million people yearly), are emerging clinical problems 25,26. Unfortunately, prognosis in those cancers is poor. Only 60% of patients with osteosarcoma and breast cancer survive at least 5 years from diagnosis, whereas almost no patients with pancreatic cancer survive 5 years (median survival 5.5 months) 26-28. Moreover, the treatment of cancer: chemotherapy, radiotherapy, hormonotherapy, and surgery severely decrease patients' quality of life. Among the others: myelosuppression, hepatotoxicity, renal failure, heart failure, gastrointestinal tract damage, nausea and hair loss are the most important side effect of chemotherapy 15,22,23. Therefore, novel approaches with better effectiveness and less severe side effects are needed.

The main aim of the study was to evaluate the anti-tumor and safety of AuNPs stabilized with GSH and conjugated with chemotherapeutics under *in vitro* condition. We decided to use AuNPs as it had been proven that they have a good safety profile and established a role as a drug delivery platform 29,30. Furthermore, our nanoparticles are coated with GSH in order to increase biocompatibility 13. Indeed, we have shown that AuNPs-GSH had small effects on the viability of mammalian cells. Importantly, typically AuNPs conjugated with chemotherapeutics have a higher impact on the viability of cancer cell lines than non-transformed ones. Furthermore, in some concentrations of AuNPs-chemotherapeutics, we were able to show selective cytotoxicity. Differences in effects exerted by AuNPs-chemotherapeutic and chemotherapeutic alone may be due to a different way of internalization. The drug can be internalized by passive diffusion whereas when conjugated with AuNPs internalization mechanism is endocytosis or other active transport mechanisms 31. More importantly AuNPs-chemotherapeutic conjugate, in some cases, were more effective than chemotherapeutic alone. We compared the cytotoxicity of chemotherapeutics on cell lines used in this study (143B, PANC-1, MCF7) with literature data, and generally, we had similar results 31,32,41,42,33-40. For DOX, Kamba et al., reported that IC_50_ was around 0.5µg/mL (MG63, osteosarcoma cells), Yui et al, showed that IC_50_ was approximately equal 2 µg/mL (PANC1 cells).

Data on cytotoxicity of AuNPs-GSH or AuNPs conjugated with chemotherapeutics are limited. However, several studies have proven the safety of AuNPs. Leite et al, have demonstrated that 4.5 nm PEG-AuNPs in concentration up to 5 × 10^13^ particles/mL did not influence the viability of mouse myoblastoma (C2C12 cells) measured by MTT assay 43. Similarly, IC_50_ of HeLa cells treated with 1.4 nm GSH-coated AuNPs was 3130 µM. In our study, which was similar to already published data, AuNPs-GSH had the lowest cytotoxicity potential, which have proven that functionalization of AuNPs with GSH ensures good safety-profile of synthesized AuNPs. Size of AuNPs is one of the main factors impacting AuNPs cytotoxicity 44. Pan et al have examined the cytotoxicity of spherical AuNPs against human cell lines (fibroblast, melanoma, epithelial cells, and macrophages). They have chosen AuNPs in size range between 0.8 and 15 nm 44. 1.4 nm AuNPs were the most cytotoxic whereas 15 nm AuNPs were 60-100 times less toxic 44.

Manivasagan et al. assessed potential anti-cancer AuNPs-fucoidan-DOX on human breast adenocarcinoma cells (MDA-MB-231). Similarly to our results they have shown that AuNPs coated with DOX had a higher impact on cancer cells viability than AuNPs-fucoidan or DOX alone 45. Correspondingly, Venkatourwar et al., assessed the impact of porphyrin-coated AuNPs conjugated with DOX as a potential drug delivery platform. They also showed that conjugated DOX and AuNPs are more cytotoxic against human glioma cells (LN-299) than any of the compounds alone 46.

According to best of our knowledge, it is the first study to examine the impact of CTA conjugated metal nanoparticles on mammalian cells in which we indicated that AuNPs stabilized with GSH and conjugated with CTA can be more effective in inducing cell death that CTA alone on osteosarcoma, pancreatic cancer cells breast cancer cells.

We have found that AuNPs may be an interesting drug delivery platform. AuNPs-chemotherapeutic conjugates may be more effective than the drug alone and can have a selective effect only on cancer cells. AuNPs-chemotherapeutic conjugates allow using lesser concentration (dose) of the drug, which decreases the severity of side effects and reduces the treatment cost.

## Conclusions

In the presented study, we demonstrated the anti-cancer potential of AuNPs stabilized with GSH and conjugated with chemotherapeutics. We have shown that our nanoparticles can be selectively cytotoxic to cancer cell lines (in comparison to non-transformed ones). Furthermore, in some cases, synthesized AuNPs conjugates were more effective than the drug alone. Modern methods of chemical synthesis of nanoparticles conjugated with chemotherapeutics may increase the effectiveness of anti-cancer therapy. At the same time, it allows for a significant reduction of treatment costs and relieves of side effects.

## Abbreviations

GEM: gemcitabine
DOX: doxorubicin
CTA: cytarabine
NPs: nanoparticles
AuNPs: gold nanoparticles
GSH: glutathione
HAuCl_4_: chloroauric acid
TEM: transmission electron microscopy
TGA: thermogravimetric analysis
NMR: nuclear magnetic resonance spectroscopy
HPLC: high-performance liquid chromatography
